# Supplementary figures and images for: Pharmacokinetic Study of Islatravir and Etonogestrel Implants in Macaques
Source: Pharmaceutics. 2023 Nov 26;15(12):2676. doi: 10.3390/pharmaceutics15122676 (PMC10747562; doi:10.3390/pharmaceutics15122676)

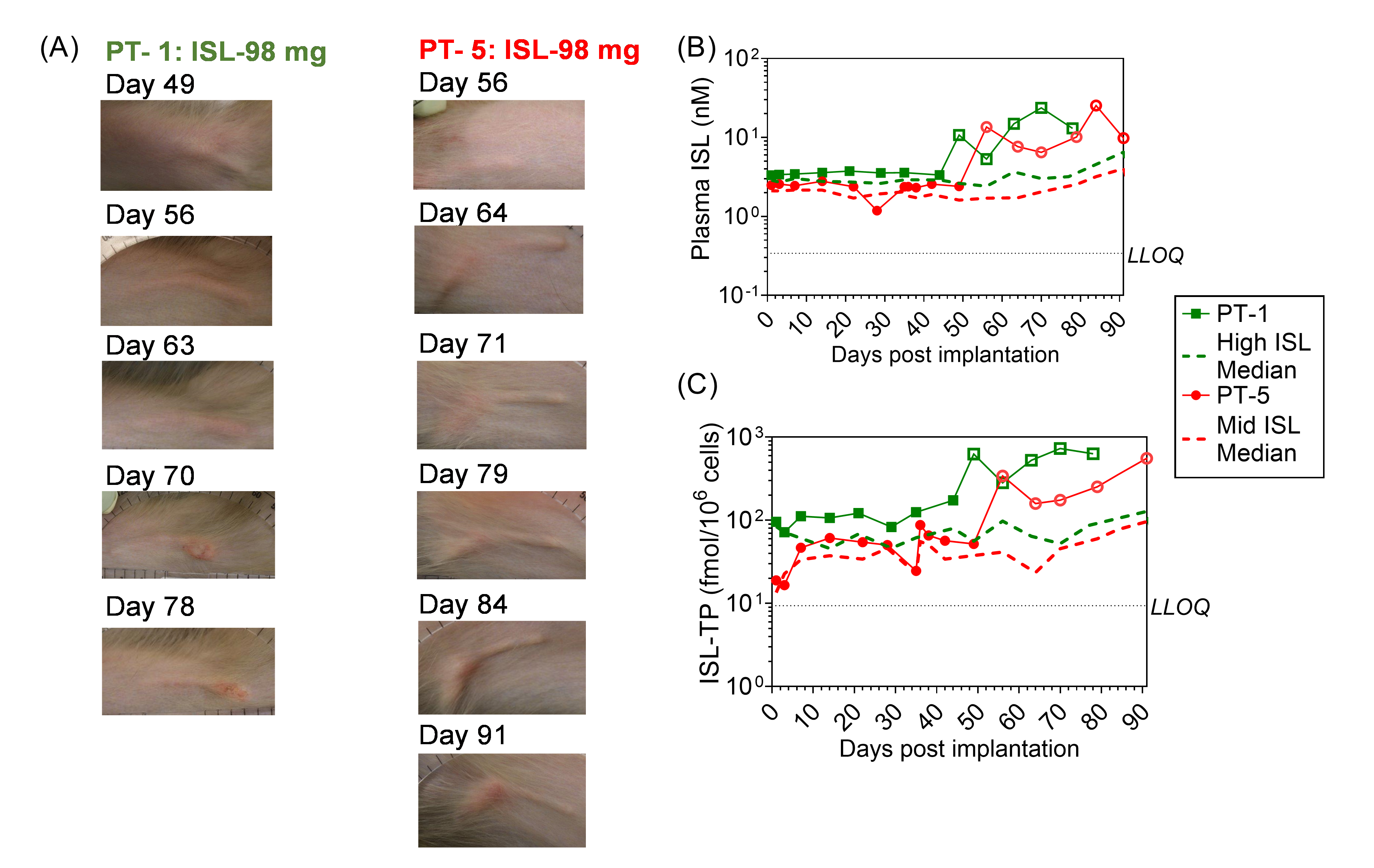

Supplement: Supplementary file 1 [file pharmaceutics-15-02676-s001.zip › Supplemental Figure S1.TIF]

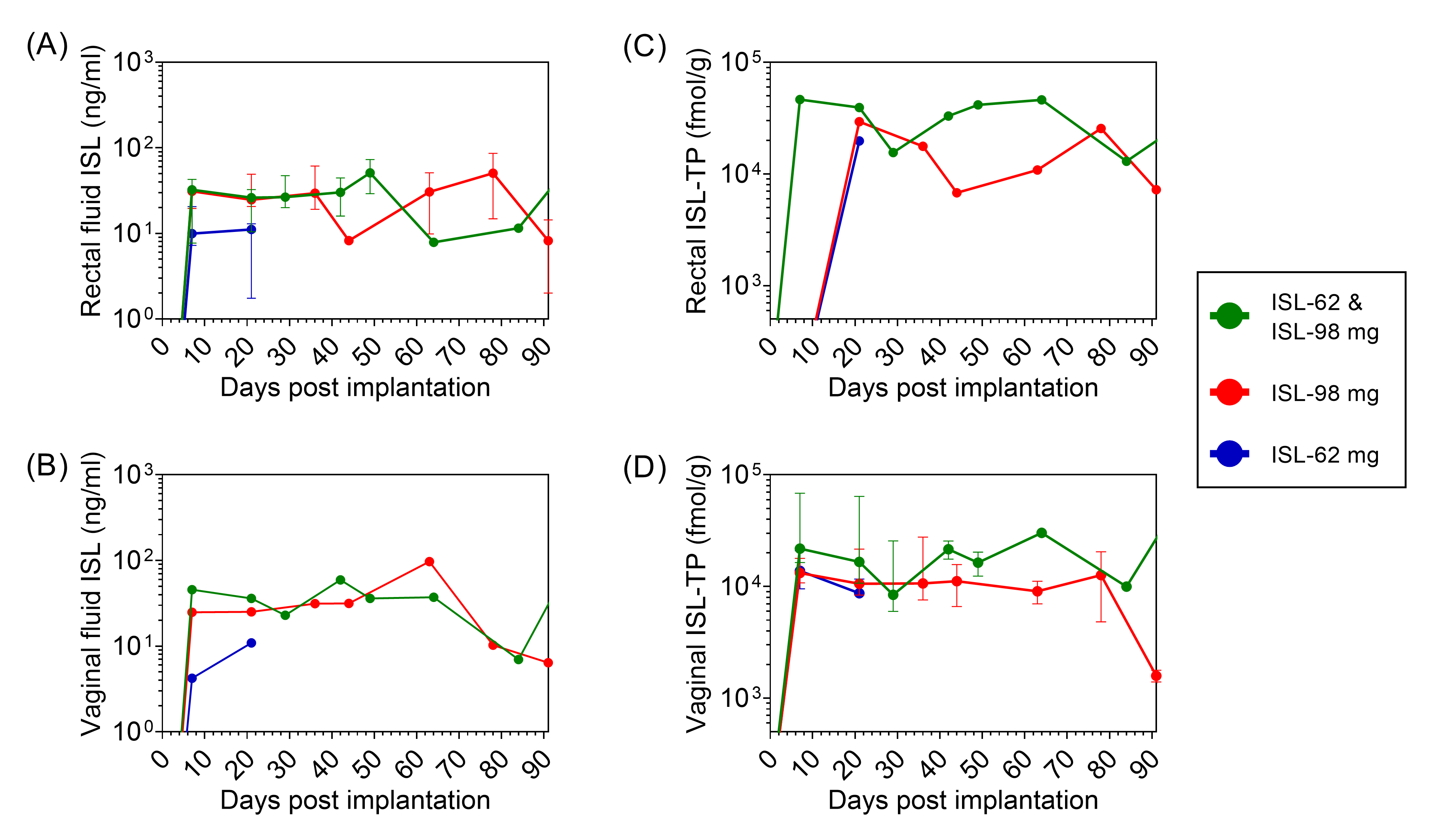

Supplement: Supplementary file 1 [file pharmaceutics-15-02676-s001.zip › Supplemental Figure S2.TIF]
